# Supplementary material for: Combining simple blood tests to identify primary care patients with unexpected weight loss for cancer investigation: Clinical risk score development, internal validation, and net benefit analysis
Source: PLoS Med. 2021 Aug 31;18(8):e1003728. doi: 10.1371/journal.pmed.1003728 (PMC8407560; doi:10.1371/journal.pmed.1003728)
Supplement: S1 Fig — (DOCX) [file pmed.1003728.s002.docx]

**S1 Fig:** Multiple imputation diagnostics for the first twenty imputations for continuous blood test variables.

**
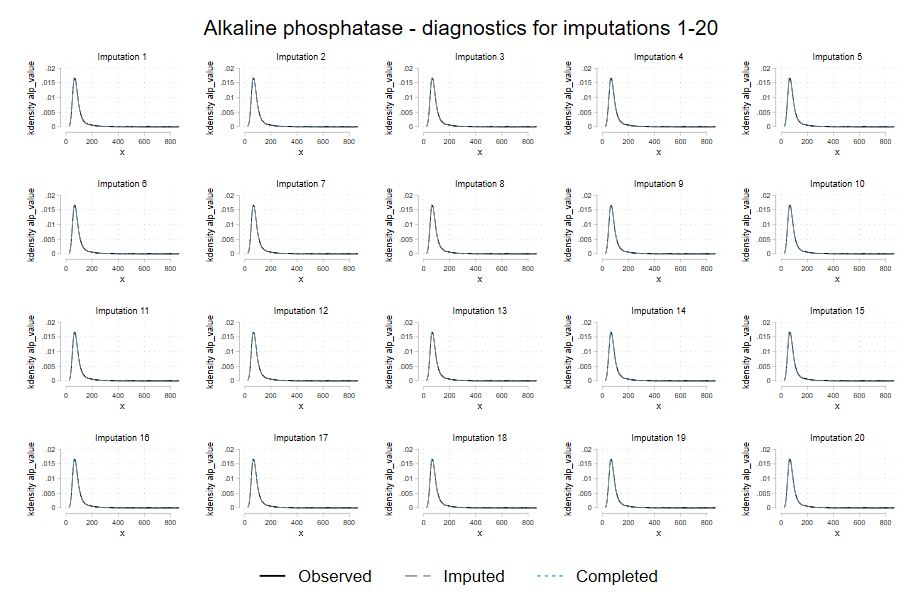

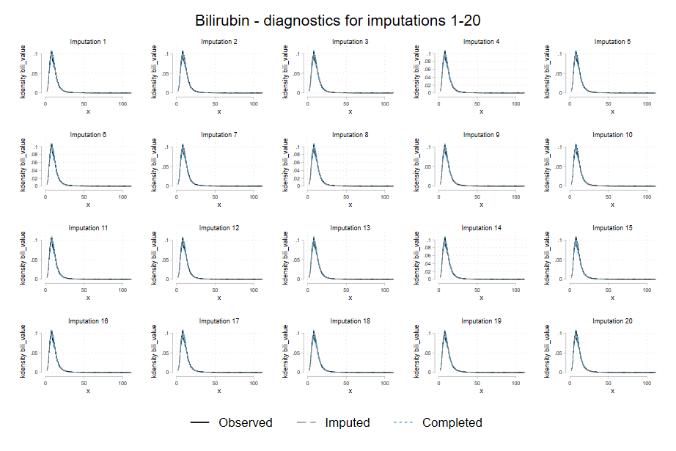

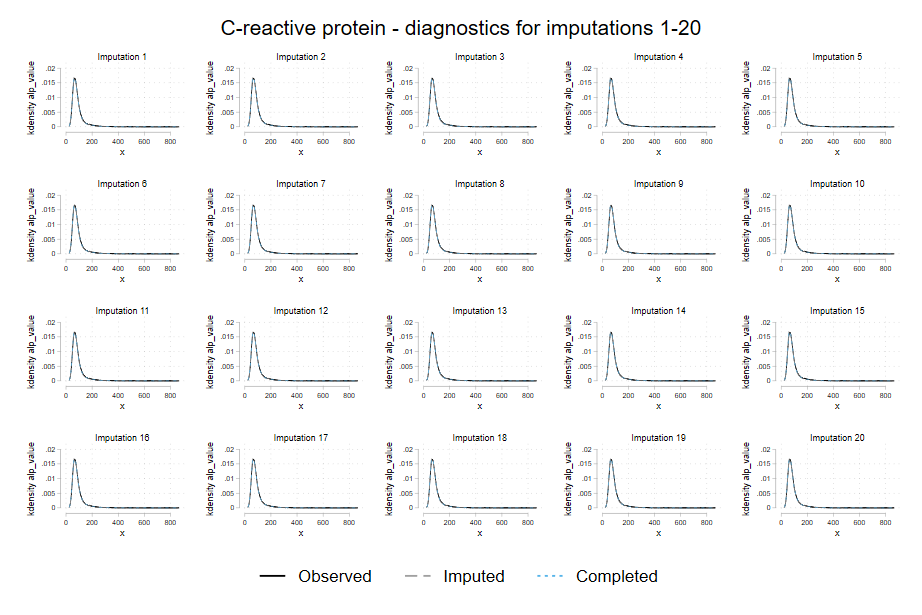

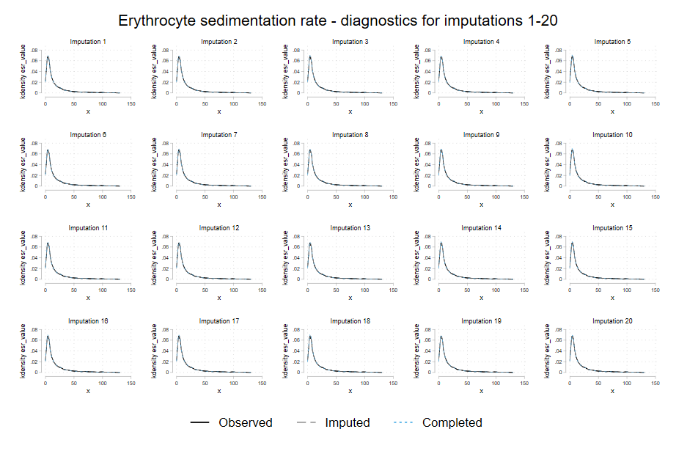

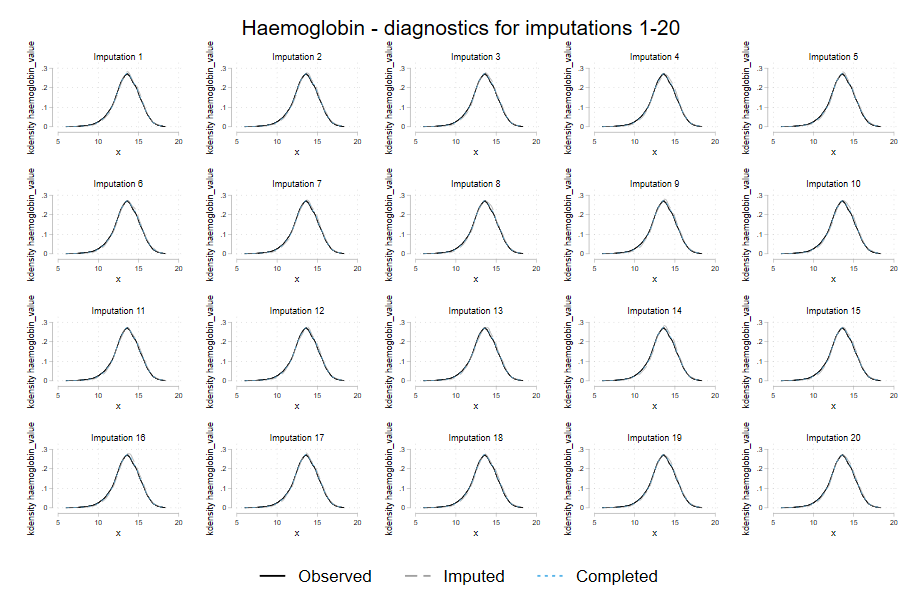

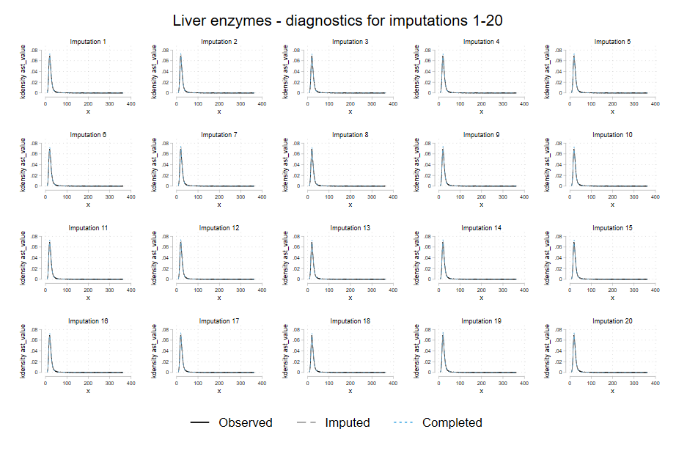

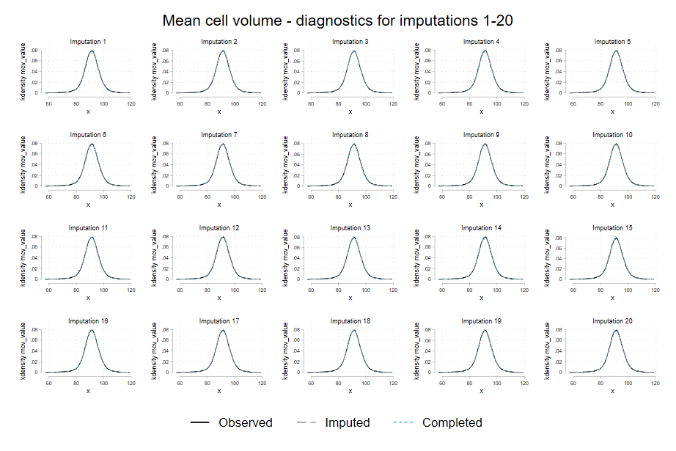

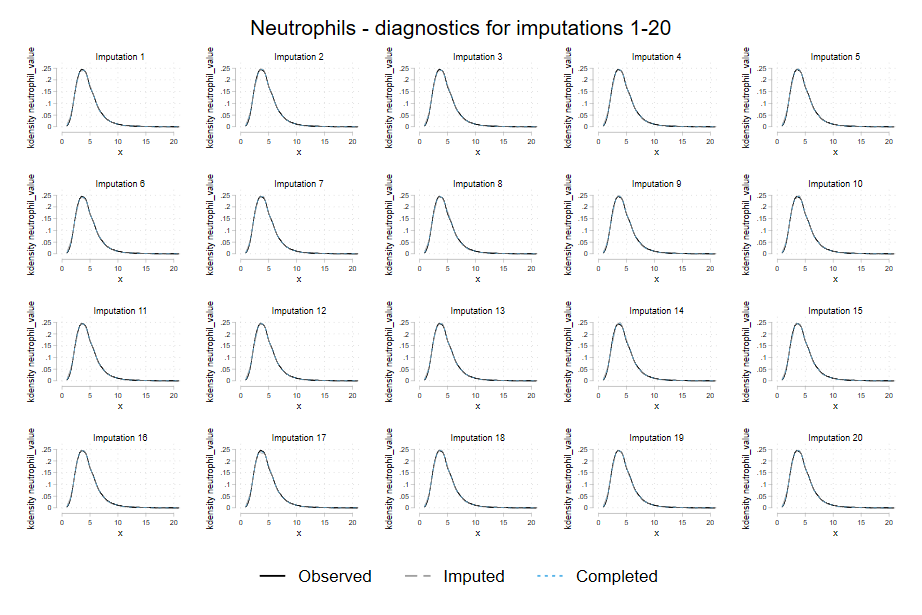

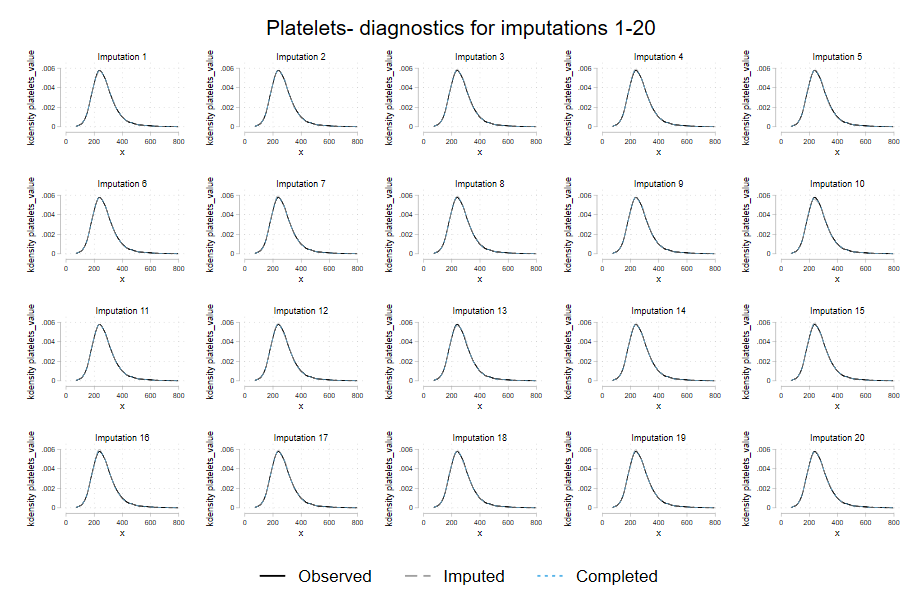
**
