# Supplementary material for: Combining simple blood tests to identify primary care patients with unexpected weight loss for cancer investigation: Clinical risk score development, internal validation, and net benefit analysis
Source: PLoS Med. 2021 Aug 31;18(8):e1003728. doi: 10.1371/journal.pmed.1003728 (PMC8407560; doi:10.1371/journal.pmed.1003728)
Supplement: S2 Fig — Green points are deciles of predicted probability with error bars. The right hand panels are zoomed in to show in detail the first 0.1% of predicted probability and observed frequency. AUC, area under the curve; CITL, calibration in the large; E:O, ratio of expected (predicted) probability vs observed frequency of the outcome; STm, symptoms and tests model; Tm, tests-only model. (DOCX) [file pmed.1003728.s003.docx]

**S2 Fig: Cross-validation plots for continuous symptoms and tests model (STm) and the tests only model (Tm).** Green points are deciles of predicted probability with error bars. The right hand panels are zoomed in to show in detail the first 0.1% of predicted probability and observed frequency.

**
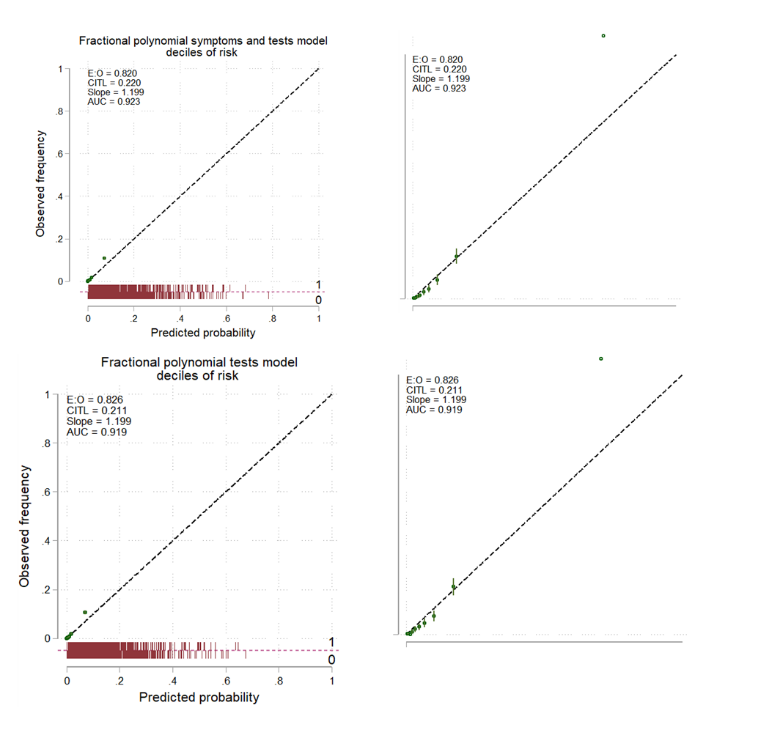
**
