# Supplementary material for: Combining simple blood tests to identify primary care patients with unexpected weight loss for cancer investigation: Clinical risk score development, internal validation, and net benefit analysis
Source: PLoS Med. 2021 Aug 31;18(8):e1003728. doi: 10.1371/journal.pmed.1003728 (PMC8407560; doi:10.1371/journal.pmed.1003728)
Supplement: S1 Table — (DOCX) [file pmed.1003728.s004.docx]

**S1 Table: Common laboratory test ranges in general practice [1].**

| Blood test group | Blood test (units) | Normal range / threshold | Abnormality associated with cancer diagnosis |
| --- | --- | --- | --- |
| Liver function tests | Albumin (g/L) | 35-50 | Low [2] |
|  | Alkaline Phosphatase (iu/L) | 30-130 | Raised [3,4] |
|  | Liver enzymes (AST /ALT) (iu/L) | 5-35 | Raised [3,4] |
|  | Bilirubin (umol/L) | 3-17 | Raised [3,4] |
|  |  |  |  |
|  |  |  |  |
| Full blood count | Haemoglobin (g/L) | 130-180 (male) 115-160 (female) | Low [5] |
|  | Lymphocytes (x10^9^/L) | 1.0-4.5 (NLS score is <1.5) | Low [6] |
|  | Mean cell volume (fL) | 76-96 | Low |
|  | Monocytes (x10^9^/L) | 0.2-0.8 | Raised [6] |
|  | Neutrophils (x10^9^/L) | 2.0-7.5 | Raised [6] |
|  | Platelets (x10^9^/L) | 150-400 | Raised [7] |
|  | Total white cell count (x10^9^/L) | 4.0-11.00 | Raised |
|  |  |  |  |
| Inflammatory markers | CRP (mg/L) | <10 | Raised [8] |
|  | Erythrocyte Sedimentation Rate (mm/h) | 0-10 (aged <65 years, male)  0-15 (aged <65 years, female)  0-20 (aged ≥65 years, male)  0-25 (aged ≥65 years, female) | Raised [8] |

**References.**

1. Simon C, Burkes M, Everitt H, van Dorp F. Oxford Handbook of General Practice Oxford: Oxford University Press; 2014.

2. Merriel SW, Carroll R, Hamilton F, Hamilton W. Association between unexplained hypoalbuminaemia and new cancer diagnoses in UK primary care patients. Family practice. 2016;33(5):449-52. Epub 2016/06/28. doi: 10.1093/fampra/cmw051. PubMed PMID: 27343860.

3. Smellie WS, Forth J, Ryder S, Galloway MJ, Wood AC, Watson ID. Best practice in primary care pathology: review 5. J Clin Pathol. 2006;59(12):1229-37. Epub 2006/04/29. doi: 10.1136/jcp.2006.037754. PubMed PMID: 16644875; PubMed Central PMCID: PMCPMC1860526.

4. Newsome PN, Cramb R, Davison SM, Dillon JF, Foulerton M, Godfrey EM, et al. Guidelines on the management of abnormal liver blood tests. Gut. 2018;67(1):6-19. Epub 2017/11/11. doi: 10.1136/gutjnl-2017-314924. PubMed PMID: 29122851; PubMed Central PMCID: PMCPMC5754852.

5. Hamilton W, Lancashire R, Sharp D, Peters TJ, Cheng KK, Marshall T. The importance of anaemia in diagnosing colorectal cancer: a case-control study using electronic primary care records. Br J Cancer. 2008;98(2):323-7. Epub 2008/01/26. doi: 10.1038/sj.bjc.6604165. PubMed PMID: 18219289; PubMed Central PMCID: PMCPMC2361444.

6. Dolan RD, McSorley ST, Park JH, Watt DG, Roxburgh CS, Horgan PG, et al. The prognostic value of systemic inflammation in patients undergoing surgery for colon cancer: comparison of composite ratios and cumulative scores. Br J Cancer. 2018;119(1):40-51. Epub 2018/05/24. doi: 10.1038/s41416-018-0095-9. PubMed PMID: 29789606; PubMed Central PMCID: PMCPMC6035216.

7. Bailey SE, Ukoumunne OC, Shephard EA, Hamilton W. Clinical relevance of thrombocytosis in primary care: a prospective cohort study of cancer incidence using English electronic medical records and cancer registry data. The British journal of general practice : the journal of the Royal College of General Practitioners. 2017;67(659):e405-e13. Epub 2017/05/24. doi: 10.3399/bjgp17X691109. PubMed PMID: 28533199; PubMed Central PMCID: PMCPMC5442956.

8. Watson J, Salisbury C, Banks J, Whiting P, Hamilton W. Predictive value of inflammatory markers for cancer diagnosis in primary care: a prospective cohort study using electronic health records. Br J Cancer. 2019;120(11):1045-51. Epub 2019/04/25. doi: 10.1038/s41416-019-0458-x. PubMed PMID: 31015558.
