# Supplementary material for: Combining simple blood tests to identify primary care patients with unexpected weight loss for cancer investigation: Clinical risk score development, internal validation, and net benefit analysis
Source: PLoS Med. 2021 Aug 31;18(8):e1003728. doi: 10.1371/journal.pmed.1003728 (PMC8407560; doi:10.1371/journal.pmed.1003728)
Supplement: S2 Table — (DOCX) [file pmed.1003728.s005.docx]

**S2 Table:** Full specification of the continuous symptoms and tests model (STm) and the test only model (Tm).

**STm**

| Covariate | Cat/form. | Coefficient (95% CI) | Odds Ratio (95% CI) | p-value |
| --- | --- | --- | --- | --- |
| Demographics |  |  |  |  |
| Age-group (ref = 60-69 yrs) | 18-39 yrs | -3.312 (-4.212--2.412) | 0.036 (0.015-0.09) | <0.01 |
|  | 40-49 yrs | -1.616 (-2.099--1.134) | 0.199 (0.123-0.322) | <0.01 |
|  | 50-59 yrs | -0.939 (-1.278--0.6) | 0.391 (0.279-0.549) | <0.01 |
|  | 70-79 yrs | 0.396 (0.183-0.608) | 1.485 (1.201-1.837) | <0.01 |
|  | 80+ yrs | 0.403 (0.182-0.623) | 1.496 (1.2-1.865) | <0.01 |
| Sex (ref = female) | Male | 0.671 (0.514-0.829) | 1.957 (1.671-2.29) | <0.01 |
| Smoking status (ref = never) | Current | 0.622 (0.369-0.876) | 1.863 (1.446-2.401) | <0.01 |
|  | Ex-smoker | 0.202 (-0.044-0.447) | 1.223 (0.957-1.564) | 0.12 |
| *Symptoms* |  |  |  |  |
| Abdominal pain | Yes | 0.618 (0.386-0.849) | 1.855 (1.471-2.338) | <0.01 |
| Change in bowel habit | Yes | 0.689 (0.209-1.169) | 1.991 (1.232-3.218) | <0.01 |
| Dyspepsia | Yes | 0.514 (0.195-0.833) | 1.672 (1.216-2.301) | <0.01 |
| *Signs* |  |  |  |  |
| Iron Deficiency Anaemia | Yes | 0.074 (-0.321-0.469) | 1.077 (0.726-1.599) | 0.71 |
| Jaundice | Yes | 0.514 (-0.157-1.185) | 1.672 (0.855-3.272) | 0.13 |
| *Blood tests (continuous)* |  |  |  |  |
| Albumin | linear | -0.026 (-0.044--0.007) | 0.975 (0.957-0.993) | <0.01 |
| Alkaline phosphatase | FP1(-0.5) | -1.542 (-1.939--1.145) | 0.214 (0.144-0.318) | <0.01 |
| Liver enzymes | linear | 0.006 (0.003-0.009) | 1.006 (1.003-1.009) | <0.01 |
| C-reactive protein | FP1(0) | 0.525 (0.432-0.617) | 1.69 (1.54-1.854) | <0.01 |
| Haemoglobin | linear | -0.063 (-0.115--0.01) | 0.939 (0.891-0.99) | 0.02 |
| Mean cell volume | linear | -0.049 (-0.062--0.037) | 0.952 (0.94-0.964) | <0.01 |
| Platelets | linear | 0.001 (0-0.001) | 1.001 (1-1.001) | 0.16 |
| Monocytes | FP1(0) | 0.355 (0.178-0.532) | 1.426 (1.195-1.702) | <0.01 |
| Lymphocytes | FP1(3) | 1.37 (-0.869-3.608) | 3.935 (0.42-36.909) | 0.23 |
|  |  |  |  |  |
| *Constant* |  | 1.195 (-0.206-2.595) |  | 0.09 |

**Tm**

| Covariate |  | Coefficient (95% CI) | Odds Ratio (95% CI) | p-value |
| --- | --- | --- | --- | --- |
| *Demographics* |  |  |  |  |
| Age-group (ref = 60-69 yrs) | 18-39 yrs | -3.137 (-4.034--2.24) | 0.043 (0.018-0.106) | <0.01 |
|  | 40-49 yrs | -1.527 (-2.006--1.049) | 0.217 (0.135-0.35) | <0.01 |
|  | 50-59 yrs | -0.898 (-1.233--0.563) | 0.407 (0.291-0.57) | <0.01 |
|  | 70-79 yrs | 0.315 (0.108-0.523) | 1.371 (1.114-1.687) | <0.01 |
|  | 80+ yrs | 0.226 (0.015-0.437) | 1.253 (1.015-1.548) | 0.04 |
| Sex (ref = female) | male | -3.137 (-4.034--2.24) | 0.043 (0.018-0.106) | <0.01 |
| *Blood tests* |  |  |  |  |
| Albumin | Linear | -0.022 (-0.04--0.004) | 0.978 (0.961-0.996) | 0.02 |
| Alkaline phosphatase | FP1(-0.5) | -1.598 (-1.989--1.208) | 0.202 (0.137-0.299) | <0.01 |
| Liver enzymes | Linear | 0.006 (0.003-0.008) | 1.006 (1.003-1.008) | <0.01 |
| C-reactive protein | FP1(0) | 0.532 (0.441-0.624) | 1.703 (1.554-1.867) | <0.01 |
| Total white cell count | Linear | 0.052 (0.027-0.077) | 1.053 (1.027-1.08) | <0.01 |
| Platelets | Linear | 0.001 (0-0.002) | 1.001 (1-1.002) | 0.01 |
| Haemoglobin | Linear | -0.11 (-0.159--0.062) | 0.895 (0.853-0.94) | <0.01 |
|  |  |  |  |  |
| Constant |  | -2.929 (-3.85--2.008) |  | <0.01 |
