# Supplementary material for: Combining simple blood tests to identify primary care patients with unexpected weight loss for cancer investigation: Clinical risk score development, internal validation, and net benefit analysis
Source: PLoS Med. 2021 Aug 31;18(8):e1003728. doi: 10.1371/journal.pmed.1003728 (PMC8407560; doi:10.1371/journal.pmed.1003728)
Supplement: S3 Table — SNB = net benefit / prevalence. SNB, standardised net benefit; STm, symptoms and test model; Sm, symptoms-only model; Tm, tests-only model. (DOCX) [file pmed.1003728.s006.docx]

**S3 Table. Standardised net benefit and reduction in further investigation comparing models and investigating all patients or none.**

STm = Symptoms and test model, Sm = Symptoms only model, Tm = Tests only model. Standardised Net Benefit = net benefit/prevalence

| **Threshold probability (%)** | **Standardised Net benefit (%)** | | | | | | | **Reduction in investigation (%)** | | | | | |
| --- | --- | --- | --- | --- | --- | --- | --- | --- | --- | --- | --- | --- | --- |
|  | **All** | **None** | **Sm** | **STm** | **Tm** | **STm - Sm** | **Tm - Sm** | **Sm** | **STm** | **Tm** | **STm - Sm** | **Tm - Sm** |  |
| 0.1 | 92.9 | 0 | 92.9 | 92.9 | 92.9 | 0 | 0 | 3.4 | 15.1 | 12.1 | 11.7 | 8.7 |  |
| 0.2 | 85.7 | 0 | 92.9 | 92.9 | 92.9 | 0 | 0 | 14.3 | 25.0 | 24.8 | 10.7 | 10.5 |  |
| 0.3 | 78.6 | 0 | 85.7 | 85.7 | 85.7 | 0 | 0 | 23.2 | 33.0 | 30.4 | 9.8 | 7.2 |  |
| 0.4 | 71.4 | 0 | 78.6 | 85.7 | 85.7 | 7.1 | 0 | 26.1 | 39.1 | 33.3 | 13 | 7.2 |  |
| 0.5 | 64.3 | 0 | 78.6 | 78.6 | 78.6 | 0 | 7.1 | 28.2 | 43.2 | 38.8 | 15 | 10.6 |  |
| 0.6 | 57.1 | 0 | 71.4 | 78.6 | 78.6 | 7.1 | 7.1 | 30.4 | 46.4 | 44.6 | 16 | 14.3 |  |
| 0.7 | 50 | 0 | 64.3 | 78.6 | 78.6 | 14.3 | 7.1 | 31.9 | 48.7 | 46.8 | 16.8 | 14.9 |  |
| 0.8 | 42.9 | 0 | 64.3 | 71.4 | 71.4 | 7.1 | 7.1 | 34.0 | 51.0 | 49.6 | 17 | 15.6 |  |
| 0.9 | 35.7 | 0 | 57.1 | 71.4 | 71.4 | 14.3 | 14.3 | 35.4 | 53.1 | 52.1 | 17.7 | 16.7 |  |
| 1.0 | 28.6 | 0 | 57.1 | 71.4 | 71.4 | 14.3 | 14.3 | 36.7 | 55.2 | 53.5 | 18.5 | 16.8 |  |
| 2.0 | -42.9 | 0 | 28.6 | 57.1 | 57.1 | 28.6 | 21.4 | 49.5 | 67.4 | 66.2 | 17.9 | 16.7 |  |
| 3.0 | -114.3 | 0 | 21.4 | 42.9 | 42.9 | 21.4 | 21.4 | 61.2 | 73.4 | 72.5 | 12.2 | 11.3 |  |
| 4.0 | -192.9 | 0 | 7.1 | 35.7 | 35.7 | 28.6 | 28.6 | 67.1 | 77.3 | 76.7 | 10.2 | 9.6 |  |
| 5.0 | -271.4 | 0 | 7.1 | 35.7 | 28.6 | 28.6 | 21.4 | 73.0 | 80.2 | 79.5 | 7.2 | 6.5 |  |
| 6.0 | -350 | 0 | 0 | 28.6 | 21.4 | 28.6 | 21.4 | 77.0 | 82.4 | 81.7 | 5.4 | 4.8 |  |
| 7.0 | -428.6 | 0 | 0 | 21.4 | 21.4 | 21.4 | 21.4 | 80.0 | 84.1 | 83.5 | 4.1 | 3.5 |  |
| 8.0 | -514.3 | 0 | 0 | 21.4 | 14.3 | 21.4 | 14.3 | 82.4 | 85.5 | 84.9 | 3.1 | 2.5 |  |
| 9.0 | -592.9 | 0 | 0 | 14.3 | 14.3 | 14.3 | 14.3 | 84.3 | 86.6 | 86.2 | 2.3 | 1.9 |  |
| 10.0 | -678.6 | 0 | 0 | 14.3 | 14.3 | 14.3 | 14.3 | 85.8 | 87.6 | 87.3 | 1.8 | 1.4 |  |
| 11.0 | -771.4 | 0 | 0 | 14.3 | 7.1 | 14.3 | 7.1 | 87.1 | 88.5 | 88.2 | 1.4 | 1.1 |  |
| 12.0 | -857.1 | 0 | 0 | 7.1 | 7.1 | 7.1 | 7.1 | 88.1 | 89.2 | 89.0 | 1.1 | 0.9 |  |
| 13.0 | -950 | 0 | 0 | 7.1 | 7.1 | 7.1 | 7.1 | 89.0 | 89.9 | 89.7 | 0.9 | 0.7 |  |
| 14.0 | -1042.9 | 0 | 0 | 7.1 | 7.1 | 7.1 | 7.1 | 89.8 | 90.5 | 90.3 | 0.7 | 0.5 |  |
| 15.0 | -1142.9 | 0 | 0 | 7.1 | 7.1 | 7.1 | 7.1 | 90.5 | 91.0 | 90.9 | 0.5 | 0.4 |  |
| 16.0 | -1242.9 | 0 | 0 | 7.1 | 0 | 7.1 | 7.1 | 91.1 | 91.5 | 91.4 | 0.4 | 0.3 |  |
| 17.0 | -1342.9 | 0 | 0 | 7.1 | 0 | 7.1 | 0 | 91.6 | 92.0 | 91.8 | 0.4 | 0.2 |  |
| 18.0 | -1442.9 | 0 | 0 | 7.1 | 0 | 7.1 | 0 | 92.1 | 92.4 | 92.2 | 0.3 | 0.1 |  |
| 19.0 | -1550 | 0 | 0 | 0 | 0 | 0 | 0 | 92.5 | 92.7 | 92.6 | 0.2 | 0.1 |  |
| 20.0 | -1657.1 | 0 | 0 | 0 | 0 | 0 | 0 | 92.9 | 93.1 | 93.0 | 0.2 | 0.1 |  |
