# Supplementary material for: Combining simple blood tests to identify primary care patients with unexpected weight loss for cancer investigation: Clinical risk score development, internal validation, and net benefit analysis
Source: PLoS Med. 2021 Aug 31;18(8):e1003728. doi: 10.1371/journal.pmed.1003728 (PMC8407560; doi:10.1371/journal.pmed.1003728)
Supplement: S4 Table — (DOCX) [file pmed.1003728.s007.docx]

**S4 Table:** Symptoms and tests model (STm) risk score thresholds expressed per 100,000 patients with unexpected weight loss investigated.

| **STm Score** | **Per 100,000 patients with UWL investigated** | | | | **Ratio of false alarms to cancers diagnosed** | **Ratio of true negatives to cancers missed** |
| --- | --- | --- | --- | --- | --- | --- |
|  | **Cancers Diagnosed** | **False alarms** | **Cancers missed** | **Correctly spared investigation** |  |  |
| -11 | 1419 | 98581 | 0 | 0 | 69:1 | N/E |
| -10 | 1419 | 98482 | 0 | 99 | 69:1 | N/E |
| -9 | 1419 | 96314 | 0 | 2267 | 68:1 | N/E |
| -8 | 1419 | 91385 | 0 | 7196 | 64:1 | N/E |
| -7 | 1419 | 86554 | 0 | 12027 | 61:1 | N/E |
| -6 | 1419 | 83005 | 0 | 15576 | 58:1 | N/E |
| -5 | 1419 | 79259 | 0 | 19322 | 56:1 | N/E |
| -4 | 1418 | 76893 | 1 | 21688 | 54:1 | 21688:1 |
| -3 | 1417 | 73739 | 2 | 24842 | 52:1 | 12421:1 |
| -2 | 1414 | 69993 | 5 | 28588 | 50:1 | 5718:1 |
| -1 | 1409 | 65359 | 10 | 33222 | 46:1 | 3322:1 |
| 0 | 1407 | 60825 | 12 | 37756 | 43:1 | 3146:1 |
| 1 | 1399 | 55797 | 20 | 42784 | 40:1 | 2139:1 |
| 2 | 1390 | 48403 | 29 | 50178 | 35:1 | 1730:1 |
| 3 | 1368 | 40221 | 51 | 58360 | 29:1 | 1144:1 |
| 4 | 1333 | 31152 | 86 | 67429 | 23:1 | 784:1 |
| 5 | 1285 | 22970 | 134 | 75611 | 18:1 | 564:1 |
| 6 | 1225 | 16759 | 194 | 81822 | 14:1 | 422:1 |
| 7 | 1140 | 11633 | 279 | 86948 | 10:1 | 312:1 |
| 8 | 1043 | 8183 | 376 | 90398 | 8:1 | 240:1 |
| 9 | 911 | 5718 | 508 | 92863 | 6:1 | 183:1 |
| 10 | 761 | 3845 | 658 | 94736 | 5:1 | 144:1 |
| 11 | 607 | 2465 | 812 | 96116 | 4:1 | 118:1 |
| 12 | 410 | 1578 | 1009 | 97003 | 4:1 | 96:1 |
| 13 | 291 | 888 | 1128 | 97693 | 3:1 | 87:1 |
| 14 | 196 | 493 | 1223 | 98088 | 3:1 | 80:1 |
| 15 | 111 | 296 | 1308 | 98285 | 3:1 | 75:1 |
| 16 | 71 | 99 | 1348 | 98482 | 1:1 | 73:1 |
| 17 | 30 | 0 | 1389 | 98581 | 0:1 | 71:1 |
| 18 | 16 | 0 | 1403 | 98581 | 0:1 | 70:1 |
| 19 | 3 | 0 | 1416 | 98581 | 0:1 | 70:1 |
| 20+ | 0 | 0 | 1419 | 98581 | N/E | 69:1 |
