# Supplementary material for: Combining simple blood tests to identify primary care patients with unexpected weight loss for cancer investigation: Clinical risk score development, internal validation, and net benefit analysis
Source: PLoS Med. 2021 Aug 31;18(8):e1003728. doi: 10.1371/journal.pmed.1003728 (PMC8407560; doi:10.1371/journal.pmed.1003728)
Supplement: S5 Table — (DOCX) [file pmed.1003728.s008.docx]

**S5 Table: Tests only model (Tm) risk score thresholds expressed per 100,000 patients with unexpected weight loss investigated.**

| **Tm Score** | **Per 100,000 patients with UWL investigated** | | | | **Ratio of false alarms to cancers diagnosed** | **Ratio of true negatives to cancers missed** |
| --- | --- | --- | --- | --- | --- | --- |
|  | **Cancers Diagnosed** | **False alarms** | **Cancers missed** | **Correctly spared investigation** |  |  |
| -10 | 1419 | 98581 | 0 | 0 | 69:1 | N/E |
| -9 | 1419 | 90399 | 0 | 8182 | 64:1 | N/E |
| -8 | 1419 | 86061 | 0 | 12520 | 61:1 | N/E |
| -7 | 1419 | 80935 | 0 | 17646 | 57:1 | N/E |
| -6 | 1419 | 78175 | 0 | 20406 | 55:1 | N/E |
| -5 | 1418 | 77485 | 1 | 21096 | 55:1 | 21096:1 |
| -4 | 1415 | 73443 | 4 | 25138 | 52:1 | 6285:1 |
| -3 | 1415 | 71077 | 4 | 27504 | 50:1 | 6876:1 |
| -2 | 1405 | 64275 | 14 | 34306 | 46:1 | 2450:1 |
| -1 | 1401 | 60430 | 18 | 38151 | 43:1 | 2120:1 |
| 0 | 1399 | 56093 | 20 | 42488 | 40:1 | 2124:1 |
| 1 | 1384 | 50474 | 35 | 48107 | 36:1 | 1374:1 |
| 2 | 1346 | 40320 | 73 | 58261 | 30:1 | 798:1 |
| 3 | 1320 | 31448 | 99 | 67133 | 24:1 | 678:1 |
| 4 | 1259 | 21590 | 160 | 76991 | 17:1 | 481:1 |
| 5 | 1192 | 15478 | 227 | 83103 | 13:1 | 366:1 |
| 6 | 1107 | 10746 | 312 | 87835 | 10:1 | 282:1 |
| 7 | 998 | 7788 | 421 | 90793 | 8:1 | 216:1 |
| 8 | 867 | 5718 | 552 | 92863 | 7:1 | 168:1 |
| 9 | 704 | 4141 | 715 | 94440 | 6:1 | 132:1 |
| 10 | 549 | 2761 | 870 | 95820 | 5:1 | 110:1 |
| 11 | 373 | 1775 | 1046 | 96806 | 5:1 | 93:1 |
| 12 | 265 | 1085 | 1154 | 97496 | 4:1 | 84:1 |
| 13 | 173 | 690 | 1246 | 97891 | 4:1 | 79:1 |
| 14 | 91 | 296 | 1328 | 98285 | 3:1 | 74:1 |
| 15 | 45 | 99 | 1374 | 98482 | 2:1 | 72:1 |
| 16 | 24 | 99 | 1395 | 98482 | 4:1 | 71:1 |
| 17 | 11 | 0 | 1408 | 98581 | 0:1 | 70:1 |
| 18 | 3 | 0 | 1416 | 98581 | 0:1 | 70:1 |
