# Supplementary material for: Combining simple blood tests to identify primary care patients with unexpected weight loss for cancer investigation: Clinical risk score development, internal validation, and net benefit analysis
Source: PLoS Med. 2021 Aug 31;18(8):e1003728. doi: 10.1371/journal.pmed.1003728 (PMC8407560; doi:10.1371/journal.pmed.1003728)
Supplement: S1 Text — (DOCX) [file pmed.1003728.s009.docx]

**S1 Text: Multiple imputation model.**

The auxiliary variables included in the imputation model were: concurrent acute pancreatitis, ascites, bone pain, chest infection, cough, fatigue, fever, hematemesis, haematuria, headache, nausea, night sweats, pelvic pain, postmenopausal bleeding, rectal bleeding, recurrent infection, reflux, shoulder pain, shortness of breath, sore throat, steatorrhea, urinary tract infection, vomiting, and pre-existing chronic kidney disease, chronic pancreatitis, diabetes, eating disorders, inflammatory bowel disease, malabsorption, obesity, renal failure, rheumatoid arthritis, and thyroid disease.
