# Supplementary material for: Combining simple blood tests to identify primary care patients with unexpected weight loss for cancer investigation: Clinical risk score development, internal validation, and net benefit analysis
Source: PLoS Med. 2021 Aug 31;18(8):e1003728. doi: 10.1371/journal.pmed.1003728 (PMC8407560; doi:10.1371/journal.pmed.1003728)
Supplement: Attachment — Submitted filename: Response to reviews.docx [file pmed.1003728.s010.docx]

Requests from the editors:

Editor request: We believe that the case for publication in PLOS Medicine would be enhanced if you were able to include an element of external validation, as suggested by one referee.

*****author response: we agree that the manuscript would be strengthened by including external validation. We hope to secure funding so we may begin the process of accessing external data to validate these models. This will likely involve data from other health data providers in different jurisdictions. As we are not in a position to conduct external validation at this time, we have included the following paragraph in the discussion, as suggested by the reviewer:*

*“We used 10-fold internal cross-validation to derive estimates of predictive performance (1). However internal validation may produce over-optimistic estimates and so external validation remains necessary to assess the generalisability of our findings in settings, populations, and subgroups of interest (2, 3). Primary care data will be retrieved from alternative clinical systems for the same time period or from the same clinical systems for a later time period to account for variation in UK practice, from international settings with alternative approaches to weight measurement and weight loss recording and where a different spectrum of patients may consult with primary care, and in systems where similar blood tests are used with differing degrees of missingness.”*

Editor request: To your data statement, please add contact information for those wishing to access data from CPRD.

*****author response: thank you we now include the following statement:*

*“Data sharing: Data cannot be shared publicly because they are analysed under licence. Permission for data access is through the CPRD Independent Scientific Advisory Committee (ISAC, contact via* [*isac@mhra.gov.uk*](mailto:isac@mhra.gov.uk)*) for researchers who meet the criteria for access to confidential data. The data underlying the results presented in the study are available from the Clinical Practice Research Datalink (CPRD,* [*cprdenquiries@mhra.gov.uk*](mailto:cprdenquiries@mhra.gov.uk)*).“*

Editor request: To your title, please add a study descriptor following a colon, e.g., "...: A diagnostic accuracy study".

*****author response: thank you. The title now reads:*

*“Combining simple blood tests to identify primary care patients with unexpected weight loss for cancer investigation: clinical risk score development, internal validation, and net benefit analysis.”*

Editor request: In the "Methods and findings" subsection of your abstract, please quote summary demographic details for study participants, and mention the recruitment period.

*****author response: thank you. The beginning of the “Methods and findings” now reads:*

*“We used data from 63,973 adults (age: mean 59 years, standard deviation 21 years; 42% male) to predict cancer in patients with UWL recorded in a large representative UK primary care electronic health record between January 1st 2000 and December 31st 2012..”*

Editor request: Please add a new final sentence to the "Methods and findings" subsection of your abstract, which should begin "Study limitations include ..." or similar and should quote 2-3 of the study's main limitations.

*****author response: thank you the “Methods and findings” now reads*

*“Study limitations include using a retrospective routinely collected dataset, a reliance on coding to identify UWL, and missing data for some predictors.”*

Editor request: We ask you to revisit the wording of the "Conclusions" subsection of your abstract, and similar statements elsewhere in the paper, e.g., at the end of the main text, which seem to us somewhat too strong. We suggest "Our findings suggest that combinations ... could be used ..." or similar.

*****author response: thanks, we have toned this wording down so that these statements now read:*

*“Our findings suggest that combinations of simple blood test abnormalities could be used to identify patients with UWL who warrant referral for investigation, while people with combinations of normal results could be exempted from referral.”*

Editor request: After the abstract, please add a new and accessible "Author summary" section in non-identical prose. You may find it helpful to consult one or two recent research papers published in PLOS Medicine to get a sense of the preferred style.

*****author response: we have now included the following “Author summary” section*:

“Why was this study done?

- The risk of an early and late stage cancer diagnosis is increased during the 3-6 months following the first record of unexpected weight loss (UWL) in primary care. UWL presents a diagnostic challenge as it is associated with a wide range of other benign and serious conditions.
- Diagnostic strategies that avoid the harms of unnecessary invasive and costly cancer investigation are required for patients with UWL. Our research has shown that the absence of individual or pairs of co-occurring clinical features does not reduce the likelihood of cancer enough to sufficiently rule-out patients from further cancer investigation. It has also identified that primary care clinicians commonly request multiple blood tests when patients present with UWL.
- We aimed to identify whether the presence or absence of risk factors, symptoms, signs, and blood test results could be used together to rule-out more accurately the need for cancer investigation in patients with UWL.

### What did the researchers do and find?

- We analysed the electronic health records of 63,693 adults with UWL recorded between January 1st 2000 and December 31st 2012 to derive three clinical scores including symptoms, symptoms and test results, and test results, to predict the risk of cancer within six months.
- The scores including test results were highly discriminative between patients with and without cancer, were well calibrated at the levels of risk that decisions to investigate are made in primary care, and showed superior clinical utility compared to the symptoms-only model.

What do these findings mean?

- Simple scores including age-group, sex and seven simple primary care blood tests (albumin, alkaline phosphatase, C-reactive protein, haemoglobin, liver enzymes, platelets, and total white cell count) could be used to select patients with UWL who do not warrant further cancer investigation in addition to those that do.
- Further research is required to validate these scores in external datasets from settings, populations, and subgroups of interest, to understand how to maximise uptake in primary care, and to assess whether the use of this approach to cancer investigation might impact cancer outcomes”.

Editor request: Please avoid the phrase "discriminated excellently".

*****author response: apologies – we have edited to read “were highly discriminative”*

Editor request: Please substitute "sex" for "gender" where appropriate, e.g., in table 1.

*****author response: yes of course. We have substituted sex for gender throughout, male for men, and female for women.*

Editor request: Throughout the text, please format reference call-outs as follows: "... clinical practice [8,9]." (noting the absence of spaces within the square brackets).

*****author response: certainly. We have tinkered with Endnote and reformatted reference call-outs as advised.*

Editor request: Please remove the information on funding and competing interests from the end of the main text. In the event of publication, this will appear in the article metadata, via entries in the submission form.

*****author response: removed*

Editor request: Please move the information on ethics approval from the end of the main text to the Methods section.
*****author response: moved*

Editor request: In the reference list, please ensure that journal names are abbreviated correctly and consistently (e.g., "PLoS ONE", "PLoS Med.").

*****author response: we have reformatted abbreviations as advised in Endnote, which seems glitch no matter how many times we amend. We have edited these in the text also.*

Editor request: Noting references 6 & 27, please ensure that all references have full access details.

*****author response: thanks - we have checked and updated references where appropriate.*

Editor request: Please include the study protocol or prespecified analysis plan as a supplementary file, referred to in your Methods section. Please highlight analyses that were not prespecified.

*****author response: we have now referenced the study protocol in the Methods section as published in BMC Diagnostic and Prognostic Research. Apologies for this omission (Nicholson BD, Aveyard P, Hobbs FDR, Smith M, Fuller A, Perera R, et al. Weight loss as a predictor of cancer and serious disease in primary care: an ISAC-approved CPRD protocol for a retrospective cohort study using routinely collected primary care data from the UK. Diagnostic and prognostic research. 2018;2:1-. doi: 10.1186/s41512-017-0019-9. PubMed PMID: 31093551.)*

Editor request: Please include a completed checklist for the most appropriate reporting guideline, e.g., TRIPOD, labelled "S1_TRIPOD_Checklist" or similar and referred to as such in the Methods section of your main text. In the checklist, please refer to individual items by section (e.g., "Methods") and paragraph number rather than by line or page numbers, as the latter generally change in the event of publication.

*****author response: we have included the checklist as “S1_TRIPOD_Checklist” supplementary material and included items by sections and paragraph in the checklist*

Comments from the reviewers:
*** Reviewer #1:

Reviewer 1: This study aims to derive and internally validate prediction models using co-occurring risk factors, symptoms, signs and blood test data to identify clinical features that could be used together to stratify cancer risk in patients attending primary care with UWL.
Comments:
"The protocol has been published" and "We followed the TRIPOD and RECORD reporting guidelines". Can the authors please supply the protocol and reporting checklists in the supplementary material?

*****author response: thanks - we have included these as also requested by the editors above.*

Reviewer 1: "Multiple imputation was used to replace missing values for smoking status, alcohol intake, body mass index, and blood tests using the mi suite of commands in Stata (15, 16). Fifty imputed datasets were created. " Did the authors undertake any sensitivity analyses for their missing value approach?

*****author response: We took the following steps to ensure that the missing value approach we have used was as robust as possible to accommodate the MAR assumption (https://stefvanbuuren.name/fimd/sec-sensitivity.html). Firstly, we included auxiliary variables in the imputation model to explain any remaining differences that may exist in the probability of MAR. Secondly, we imputed continuous variables before dichotomising. Thirdly, we conducted a sensitivity analysis in which we built each model using an additional category for missing data (the missing indicator method) instead of including imputed data. We found that the direction of the estimates were the same and the confidence intervals overlapping when comparing the variables in the final imputed and missing indicator models. We have included the following sentences in the manuscript:*

*“We refitted the models using the missing indicator method to assess our approach to multiple imputation.” (Methods).*

*“The direction of the estimates were the same and the confidence intervals overlapping when comparing variables included in the final imputed and missing indicator models.” (Results).*

Reviewer 1: "Three prediction models were derived in the complete dataset: a symptoms only model (Sm), a symptoms and tests model (STm), and a simple tests only model (Tm). The mim Stata command was used to select variables for each model in the imputed data using backwards stepwise logistic regression, using a p-value of <0.01 for inclusion." A technically appropriate statistical approach has been applied.

*****author response: thank you*

Reviewer 1: Internal cross-validation and conducting sensitivity analyses refitting models using continuous data, are rigorous and robust methodologies.

*****author response: thank you*

Reviewer 1: Overall, this is a clearly laid out and well written study.  The authors have suitably acknowledged the main limitations, and present a thorough range of analyses in the supplementary material.

*****author response: thank you*

*** Reviewer #2:
Reviewer 2: It is important to develop predictive tools that can help clinicians select patients with unexpected weight loss for further testing. I applaud the authors for conducting this important work.

*****author response: thank you*

Reviewer 2: I however have some comments and concerns:
 1. How did you define UWL? In the first paragraph of the Methods section you write: "…12 months of data before their first UWL code (the "index date").". What is a "UWL code"? Some ICD-code? I suggest you specify.

*****author response: we have added further detail to the manuscript about the definition of UWL. The Methods section now reads:*

*“Patients were included if they were ≥18 years of age, registered with a CPRD general practice, eligible for linkage to NCRAS and Office for National Statistics (ONS) data, and at least 12 months of data before their first UWL code (the “index date”). These UWL Read codes equated to a mean weight loss of ≥5% within a 6-month period in our previous internal validation study of weight-related coding in CPRD (4). UWL may be coded following a range of clinical scenarios, including UWL reported as the patient’s presenting complaint, after targeted history taking, following weight measurement as part of the clinical examination, or as part of a routine health check or chronic disease review (5)”*

Reviewer 2: 2. For the outcome, you only consider cancer diagnosed within the first 6 months after the UWL diagnosis. This appears short to me. I see many cancer patients in my clinic for whom the diagnostic workup has been longer than 6 months. I know you found the mean time between UWL and cancer diagnosis in your previous study to be 181 days (reference 2 in your paper). I nevertheless suggest you consider to use, at least in a sensitivity analysis, 12 months or longer follow-up.

*****author response: Reference 2 reports our Br J Cancer analysis designed to explore the time following an UWL presentation for which there is an increased risk of cancer compared to a matched cohort of people not presenting with UWL. This research showed that if cancer is not diagnosed within six months, the risk of cancer being the cause of the unexpected WL is low. The full sentence from reference 2 reads “The time to diagnosis was significantly shorter in patients with unexpected weight loss (median 80 days (IQR 26–290)), with a positive skew (mean 181 days (standard deviation 205), compared to patients without (median 353 (IQR 181-541), p < 0.01). The figure shown below is also from reference 2 and demonstrates why we have elected to truncate follow-up at six months in this and a previous analysis (Reference 5: doi: 10.1136/bmj.m2651). Extending the follow-up period longer than six months would risk misattributing cancers in this dataset to the UWL presentation.*

Reviewer 2: 3. You used multiple imputation with 50 imputed datasets. For some variables, you had a very high proportion of missing (eg, 77% for CRP). Is 50 imputed datasets really enough? I suggest you motivate in your paper why you only used 50 imputed datasets.

*****author response: The proportion of missing data across smoking status, alcohol use, BMI, and the thirteen blood test variables ranges from 11.3% (BMI) to 82.9% (Liver enzymes), with the average missingness (across those variables with missing data) of 41.78%. Guidance on the optimal number of imputations varies. Van Burren (Flexible Imputation of Missing Data - 2018) states “if calculation is not prohibitive, we may set m (the number of imputations) to the average percentage of missing data. The substantive conclusions are unlikely to change as a result of raising m beyond m = 5”. At 50 imputations we are well above the average missingness. Furthermore, when developing the imputation model we started at 10 imputations and increased in tens up to 50 imputations noticing that estimates stabilised. We judged there was no further benefit of additional imputations given the increased time and computational intensity required for the imputations to complete.*

Reviewer 2: 4. You use 10-fold internal cross-validation to assess model performance. To properly internally validate the model, shouldn't you validate the whole model development process including variable selection? That is, if you use 10-fold cross validation, you should in each of the 10 runs re-select the variables, fit the model and test it in the 10th fold. And then finally average the performance. You could also consider a split-sample approach.

****author response: During final model derivation and internal cross-validation we took the following steps which required significant computation taking over three months to complete:

-we imputed fifty replicates of the dataset using predictive mean matching for continuous variables.
-we used backwards stepwise selection within each of the fifty imputed dataset and combined the results using Rubin’s rules to derive the final model.

-internal 10-fold cross-validation was then conducted for the selected model in each of the fifty imputed datasets, averaging the AUCs corresponding to each fold, which were then averaged to produce an overall model performance

Adding derivation of each model in each fold would be inadvisable as results are likely to vary significantly in the smaller samples created. Given the steps outlined above it would also be too computationally intensive. Furthermore, we are not aware of a method / command to combines the results of models derived in each fold prior to cross-validation.

Reviewer 2: 5. Why not test the performance of the previous models (reference 8, 9, 41, 42) in your data set? If you do that, you would have a chance to i) externally validate their models, and ii) benchmark your model against previous models.

*****author response: this is a good suggestion and something we considered. However, the models from refs 8 and 9 were based on data from secondary care in Spain and include Lactate Dehydrogenase (LDH), a non-specific tumour marker very uncommonly used in (UK) primary care and not included in our dataset. Models 41 and 42 are primary care cohorts but they include the consulting primary care population in general rather than only patients consulting with UWL. To externally validate models 41 and 42 would require a new application for CRPD data from the entire consulting population.*

Reviewer 2: 6. I think it is a rather major concern that your dataset/CPRD represents only 6.9% of the UK population, you have a very high proportion of missing (ie, 70-80%) for key predictors, and there are obvious concerns with over-fitting. The value and impact of your study would increase substantially with external validation. I thus strongly suggest you try to find a dataset for external validation. If this is not possible, I suggest you add a paragraph discussing these limitations and clearly state that additional research is needed before your tool can be used in clinical practice.

*****author response: we agree that external validation is necessary before implementation of these tools in clinical practice. This point is also reiterated by the Editors first comment. As mentioned above, and unfortunately, we are not in a position to conduct external validation at this time. We have followed this advice and added further details of these limitations and stated that additional research is needed before your tool can be used in clinical practice. The limitations section now reads:*

*“We used 10-fold internal cross-validation to derive estimates of predictive performance (1). However internal validation may produce over-optimistic estimates and so external validation remains necessary to assess the generalisability of our findings in settings, populations, and subgroups of interest (2, 3). Primary care data will be retrieved from alternative clinical systems for the same time period or from the same clinical systems for a later time period to account for variation in UK practice, from international settings with alternative approached to weight measurement and weight loss recording and where a different spectrum of patients may consult with primary care, and in systems where similar blood tests are used with differing degrees of missingness.”*

*** Reviewer #3:
Reviewer 3: No comments basically

*****author response: thank you*

Reviewer 3: Very well written; very important analyses, and especially the explanation box1 quite helpful for many (lay) readers

*****author response: thank you*

Reviewer 3: Should authors define UWL somewhere

*****author response: we should and we have now.*

Reviewer 3: Typing error? Age 0+ in discussion

*****author response: sorry, we are unable to find this one.*

Reviewer 3: Should results lead to clear GP guidelines ? (It is not advisable to have individual GPS make their own cut-offs

*****author response: There is not likely to be one answer to guide GPs but rather the threshold for action will be set by the health system the GP works in based on local resourcing, health economic modelling. We have illustrated how these approaches to clinical feature combination could be used in Box 1 in line with current recommendations from NICE about the threshold at which investigation is recommended (3% PPV). Box 1 also illustrates the trade-offs involved in maximising sensitivity and of balancing sensitivity and specificity.*
