# Supplementary material for: Combining simple blood tests to identify primary care patients with unexpected weight loss for cancer investigation: Clinical risk score development, internal validation, and net benefit analysis
Source: PLoS Med. 2021 Aug 31;18(8):e1003728. doi: 10.1371/journal.pmed.1003728 (PMC8407560; doi:10.1371/journal.pmed.1003728)
Supplement: Attachment — Submitted filename: Response to reviews 2.docx [file pmed.1003728.s011.docx]

Requests from Editors:

Thank you for your responses. Before we proceed, please address the following:
1.      Given the lack of external validation, please temper the phrases “excellent discrimination” and “highly discriminative”, by removing the words “excellent” and “highly.” For example, use objective terms such as “reasonably discriminative”, “discriminative” or similar.

*****author response: thanks - we have followed the example and edited throughout.*

2.      Please do not report P<0.01; report as P < 0.001.

*****author response: thanks we have now reported as P<0.001.*

3.      Please define the abbreviations in Table 4: AST, ALT, CRP

*****author response: we have provided clarification of these abbreviations in the Table legend.*

4.      Please use the "Vancouver" style for reference formatting and see our website for other reference guidelines.

*****author response: ok - we have switched the style using Endnote.*

5.      Please include a weblink and access date for ref # 4

*****author response: sure – we have updated this reference to now read*

*4. NICE. Suspected cancer: recognition and referral (NG12). National Institute for Health and Care Excellence 2015. https://www.nice.org.uk/guidance/ng12. last accessed: 14/06/2021.*

6.      Please include line numbers in your next draft

*****author response: of course – we have done this now.*
